# The Role of microRNAs and Cell-Free DNAs in Fungal Infections: Systematic Review and Meta-Analysis of the Literature

**DOI:** 10.3390/jof11100718

**Published:** 2025-10-04

**Authors:** Ayse Kalkanci, Fatma Bozdag, Isil Fidan, Ozlem Guzel Tunccan, Sultan Pinar Cetintepe, Mustafa Necmi Ilhan

**Affiliations:** 1 Department of Medical Microbiology, Faculty of Medicine, Gazi University, Ankara 06500, Türkiye; isilfidan@yahoo.com; 2 Department of Public Health, Division of Occupational Medicine, Faculty of Medicine, Gazi University, Ankara 06500, Türkiye; fatmabozdag7@gmail.com (F.B.); spinarcetintepe@gmail.com (S.P.C.); mnilhan@gazi.edu.tr (M.N.I.); 3 Department of Infectious Disease and Clinical Microbiology, Faculty of Medicine, Gazi University, Ankara 06500, Türkiye; oguzel@gazi.edu.tr

**Keywords:** fungal infections, miRNA, cfDNA, pathogenesis, diagnosis, treatment

## Abstract

Background: Invasive fungal infections (IFIs) remain a major cause of morbidity and mortality among immunocompromised patients, despite advances in antifungal therapy. Conventional diagnostics are limited, highlighting the need for novel biomarkers. Circulating microRNAs (miRNAs) and cell-free DNA (cfDNA) have emerged as promising tools due to their roles in immune regulation, pathogen–host interactions, and disease monitoring. This systematic review and meta-analysis evaluate their diagnostic and prognostic potential in fungal infections. Methods: A systematic search of PubMed, Web of Science, SCOPUS, and EMBASE was conducted up to May 2025 in line with PRISMA guidelines (PROSPERO protocol CRD42021287150). Eligible studies included clinical research on confirmed fungal infections assessing cfDNA or miRNAs. Random-effects meta-analyses were performed for cfDNA, and miRNA findings were synthesized descriptively. Results: In total, 526 studies were included. cfDNA positivity was observed in 12% of all tested samples (95% CI: 0.06–0.22) and in 79% of patients with proven fungal infections (95% CI: 0.62–0.90), supporting its value as a minimally invasive, culture-independent diagnostic marker. Six studies on miRNAs identified disease-specific signatures, including miR-132 and miRNA panels for aspergillosis, with high diagnostic accuracy (AUC ≥ 0.98). miR-146a, miR-223, and miR-545 further correlated with prognosis and mortality. Conclusions: cfDNA and miRNAs show strong potential for early diagnosis, prognosis, and treatment monitoring in IFIs. Standardized methodologies and large-scale validation are essential for clinical translation.

## 1. Introduction

Fungi are eukaryotic organisms with diverse pathogenic potential. Over 1.5 million species have been identified globally, of which a fraction have been isolated from humans. The most frequently isolated pathogens are *Candida albicans*, *Aspergillus fumigatus*, *Cryptococcus neoformans*, and species within the order Mucorales. These can cause superficial, subcutaneous, or systemic fungal infections. Systemic infections pose the greatest threat due to their association with high mortality rates, especially among immunocompromised individuals. Despite actual advanced antifungal therapies, the high morbidity and mortality rates associated with invasive fungal infections (IFIs) necessitate the approach of innovative diagnostic and therapeutic strategies [1,2,3,4,5].

This review will focus on the role of microRNAs (miRNAs) and cell-free DNAs (cfDNAs) in fungal infections from an exosome-based perspective. MicroRNAs (miRNAs) and cfDNAs are two biomolecular entities that have gained attention recently due to their importance in cellular communication and regulation of anti-microbial immune response. MicroRNAs are small, non-coding RNAs that regulate gene expression at the post-transcriptional level. They are involved in various cellular processes, including immune regulation and pathogen–host interactions [6]. Cell-free DNAs (cfDNA), on the other hand, are extracellular, ranging from 30 to 150 bp in size, and secreted by various cell types. Cell-free DNAs are essential mediators of host–pathogen interactions, similarly to miRNAs [7]. These extracellular vesicles (EVs) are formed within the endosomal system through the invagination of the endosomal membrane, resulting in multivesicular bodies (MVBs). Upon fusion of MVBs with the plasma membrane, exosomes are released into the extracellular space. Exosomes are characterized by their lipid bilayer membrane and contain diverse biomolecules, such as proteins, lipids, and nucleic acids, including RNAs and miRNAs. Exosomes are enriched with specific lipids, tetraspanins (CD63, CD81, CD9), heat shock proteins (HSP70, HSP90), and nucleic acids. Their cargo composition is highly dynamic and reflects the physiological or pathological state of the parent cell [8,9].

This review delves into the critical roles of miRNAs and cfDNAs in fungal infections, highlighting their potential as diagnostic biomarkers and therapeutic targets. Exosomes and miRNAs are closely interconnected because exosomes serve as natural carriers for miRNAs, facilitating their transfer between cells. This relationship is particularly significant in the context of fungal infections, where exosomal miRNAs play crucial roles in modulating host–pathogen interactions. Exosomes protect miRNAs from degradation in the extracellular environment, ensuring their stability and efficient delivery to target cells. Upon uptake by recipient cells, miRNAs regulate gene expression, influencing immune responses, inflammation, and cellular processes [10,11]. Studying miRNAs and cfDNAs together provides a comprehensive understanding of their synergistic roles in fungal pathogenesis and host immune modulation while also uncovering their potential as biomarkers and therapeutic agents in managing fungal infections [12].

## 2. Materials and Methods

### 2.1. The Literature Search, Selection Process and Data Extraction

**Figure 1:**
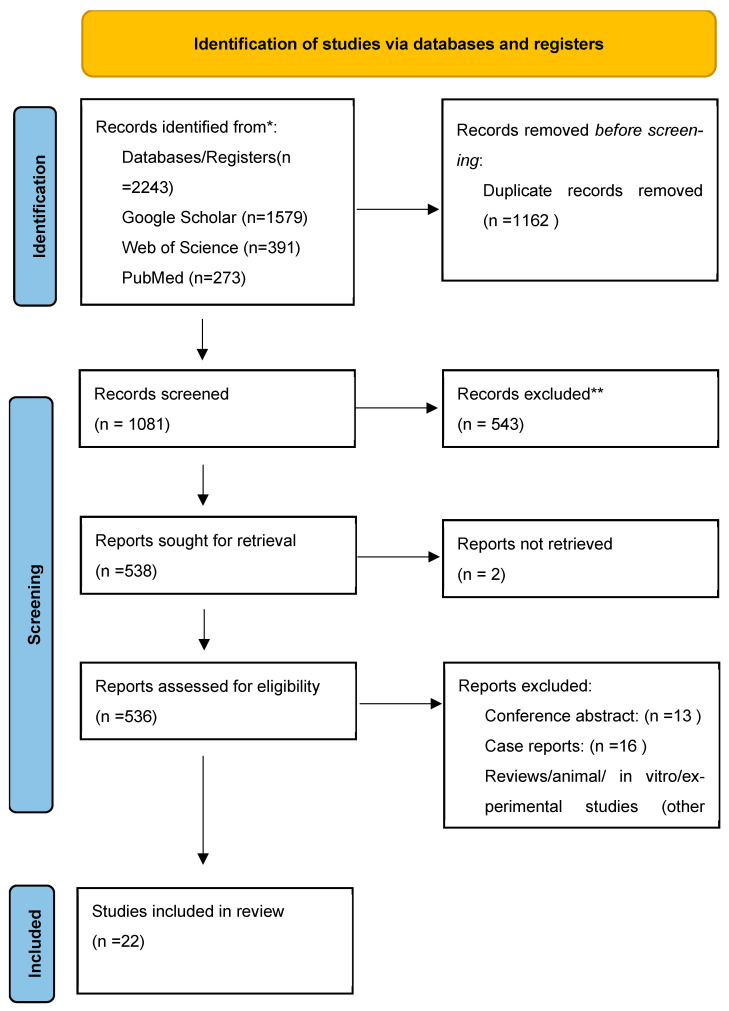
* Consider, if feasible to do so, reporting the number of records identified from each database or register searched (rather than the total number across all databases/registers). ** If automation tools were used, indicate how many records were excluded by a human and how many were excluded by automation tools. Source: [13]. This work is licensed under CC BY 4.0. To view a copy of this license, visit https://creativecommons.org/licenses/by/4.0/.

In this systematic review, the Preferred Reporting Items for Systematic Reviews and Meta-Analyses (PRISMA) methodological approach was included in the literature assessment to describe data collection and research impact. The study was registered in the International Prospective Register of Systematic Reviews (PROSPERO) on [January 2025] under protocol number [540336]. The protocol for this systematic review was also pre-registered on PROSPERO with registration number CRD42021287150. (https://app.covidence.org/reviews/540336) (accessed on 23 January 2025) Two authors (AK and IF) systematically and independently searched publications in the following sources up to end of May 2025: databases of medical literature (MEDLINE via PubMed, Web of Science, SCOPUS, and EMBASE) for relevant peer-reviewed articles. Only publications in English were included. Studies focusing on the use of miRNAs and cfDNAs as biomarkers, diagnostic studies and/or observational studies were included, while preclinical or non-human studies, studies using synthetic nanoparticles, systematic reviews, case reports and retrospective studies were excluded. The PRISMA guidelines were used to implement the literature review process. A PRISMA flow diagram was used to visualize the number of included and excluded studies and the reason behind their exclusion (Figure 1). Duplicate records from different databases were removed before screening. The initial selection was based on the title, abstract and keywords, followed by a final selection based on the full text. The patient intervention comparison outcome (PICO) scheme was applied to this study (Population: patients diagnosed with fungal infections; Intervention: cfDNA, and miRNA; Comparator: standard diagnostic methods; Outcome: prognosis, diagnosis, and treatment of fungal infections). Articles available in both English and Turkish were included in the search. Where available, the following information was extracted from the full-text articles: (i) general information (author, title, source, publication date, language, duplicate publications, enrolment dates); (ii) study characteristics (study design, duration of follow-up, and sample size); and (iii) participant characteristics (gender, age, ethnicity, number of participants recruited, disease and stage). Disagreements in selected records between reviewers (AK and IF) were solved by a consensus meeting after reading the full-text article.

### 2.2. Data Synthesis and Statistical Analysis

Two authors (FB and SPC) performed the data analysis. The review was conducted to address four main research questions. (1) In patients with fungal infections, are cfDNA and miRNA more effective than standard diagnostic methods in improving prognosis, diagnosis, and treatment outcomes? (2) Can cfDNA and miRNA serve as more reliable biomarkers than standard diagnostic methods for the early detection of fungal infections? (3) Are cfDNA and miRNA effective at predicting the prognosis of patients with fungal infections compared to standard diagnostic methods? (4) How do cfDNA and miRNA in-fluence treatment decision-making and outcomes in fungal infections compared to standard diagnostic methods? Categorical data were summarized as numbers, ratios and/or percentages calculated using Microsoft Excel 16.80. Overall proportions and corresponding 95% confidence intervals were calculated using a random-effects meta-analysis for pro-portions. Heterogeneity between studies was further explored through subgroup analyses. Subgroup differences were evaluated using between subgroups heterogeneity statistic in the random-effects meta-analysis. Heterogeneity was quantified by the percentage of variation in study estimates due to heterogeneity rather than chance. Forest plots were used to visualize the results. Since heterogeneity was detected based on the I^2^ values, a random-effects model was applied. Data analyses were conducted using R software version 4.2.2 (R Foundation for Statistical Computing, Vienna, Austria). Any *p*-value < 0.05 was considered statistically significant.

### 2.3. Final Step Analysis Protocol

In the final step of the analysis protocol, all eligible studies were synthesized, and quantitative data were pooled for meta-analysis according to the predefined methodology. Studies involved patients diagnosed with fungal infections (e.g., *Candida*, *Aspergillus*, *Cryptococcus*, etc.) using clinical, microbiological, or molecular methods; immunocompetent and immunocompromised populations (e.g., patients with cancer, HIV, and organ transplants); both adult and pediatric populations, evaluations of cfDNA and miRNA as biomarkers for the diagnosis, prognosis, and treatment of fungal infections; studies investigating cfDNA and miRNA in conjunction with other diagnostic tools; and research on the molecular mechanisms linking these biomarkers to fungal infection outcomes. The types of studies included were randomized controlled trials (RCTs), cohort studies (prospective or retrospective), case–control studies, cross-sectional studies, and systematic reviews and meta-analyses with primary data extraction.

The following were excluded from the review: animal experiments and plant modeling studies; studies on unconfirmed fungal infections or non-infectious colonization; studies that explored cfDNA and miRNA only in non-fungal infections or other diseases; studies focusing on other biomarkers unrelated to cfDNA and miRNA; and interventions not directly involving the diagnostic, prognostic, or therapeutic applications of cfDNA and miRNA. Additionally, narrative reviews, opinion pieces, editorials, and commentaries, case reports and case series with fewer than five patients, studies without full-text availability, and those not published in English were excluded. If the accession numbers were not available at the time of submission, they had to be provided during review and prior to publication.

Intervention-based studies involving animals or humans, and other studies that required ethical approval, had to state the authority that provided approval and the corresponding ethical approval code.

## 3. Results

### 3.1. Search Results

**Figure 2:**
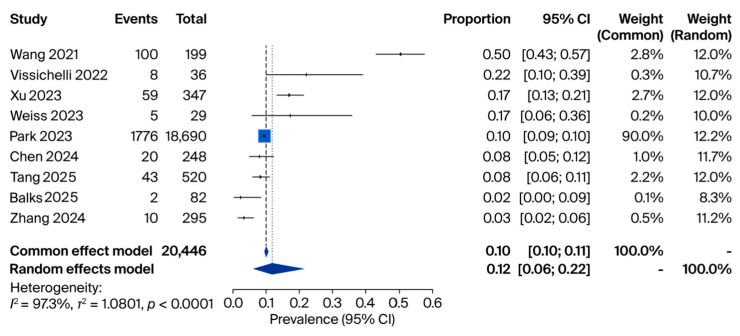
Meta-analysis of fungal positivity in cfDNA samples obtained from human subjects. Source: [12,14,15,18,19,20,21,22,23].

**Figure 3:**
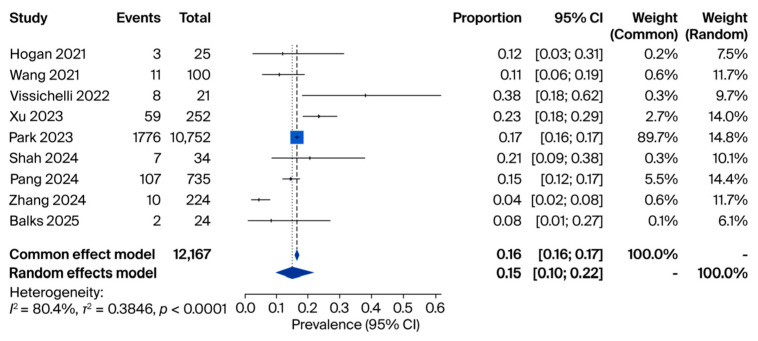
Meta-analysis of fungal positivity among cfDNA-positive samples. Source: [12,14,15,18,20,23,24,25,26].

**Figure 4:**
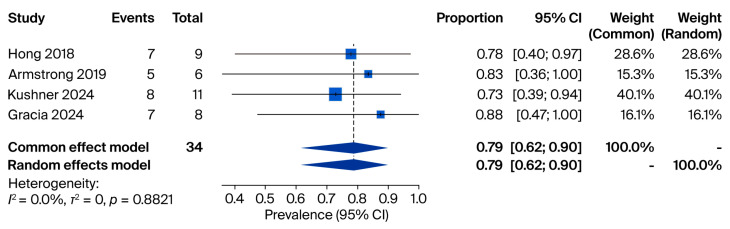
Meta-analysis of cfDNA positivity among patients with confirmed fungal infections. Source: [16,17,27,28].

We followed the approach as laid out in the PRISM flow diagram (Figure 1) to enable a systematic search of relevant databases and registers, and selected interventional clinical studies that complied with the predefined inclusion and exclusion criteria. A total of 2243 records were identified through database searches, including 1579 records from Google Scholar, 391 records from Web of Science, and 273 records from PubMed. After removing 1162 duplicates identified by Covidence, 1081 records remained for screening. During the initial screening phase, 12 studies were excluded due to an incorrect patient population. The full texts of 538 articles were assessed for eligibility, resulting in the exclusion of 543 studies that did not meet the inclusion criteria. No additional studies were retrieved from other sources, citation searching, or gray literature. Ultimately, 526 studies met the eligibility requirements and were included in the systematic review. No studies were awaiting classification or still ongoing at the time of the analysis (23 January 2025). Of the 526 studies screened, 16 cfDNA studies were ultimately included in the quantitative synthesis and provided the data for the pooled analyses shown in Figure 2, Figure 3 and Figure 4. For miRNAs, six eligible studies were included and descriptively synthesized due to heterogeneity in study design and out-comes, as summarized in Table 1.

### 3.2. cfDNA Analyses

For studies evaluating fungal positivity in cfDNA samples obtained from human subjects, eight eligible studies with a total of 20,446 samples were included in the meta-analysis. In the first analysis, fungal DNA positivity was assessed across all tested cfDNA samples (n = 20,446). Reported prevalence rates varied between 3% [12] and 50% [14], and the pooled prevalence was 12% (95% CI: 0.06–0.22) using a random-effects model (Figure 2).

In the second analysis, we restricted the evaluation to cfDNA-positive samples only (n = 12,167) to determine the proportion attributable specifically to fungal DNA. Here, individual study sizes ranged from 21 to 10,752, with prevalence rates ranging from 4% [12] to 38% [15]. The pooled prevalence was 15% (95% CI: 0.10–0.22) (Figure 3).

In the third analysis, cfDNA positivity was examined among patients with confirmed fungal infections. Four studies comprising 34 patients (sample sizes 6–11) met the inclusion criteria. Reported prevalence rates ranged from 73% [16] to 88% [17], and both fixed- and random-effects models yielded a pooled prevalence of 79% (95% CI: 0.62–0.90) (Figure 4).

**Table 1:** Summary of studies investigating miRNAs in fungal and related infections [29,30,31,32,33,34].

Study (Year)	Country	Design	Sample Size	miRNA(s) Investigated	Main Finding	Diagnostic Performance
Wang 2010 [29]	China	Case–control	Sepsis: 50, SIRS: 30	miR-146a, miR-223, miR-126, miR-15b, miR-132, miR-155, and let-7i	miR-146a and miR-223 significantly lower in sepsis; miR-126 lower in sepsis and SIRS vs. controls	miR-223: AUC 0.858 (Sens 80%, Spec 100%); miR-146a: AUC 0.804 (Sens 63.3%, Spec 100%)
Da Lacorta Singulani 2017 [30]	Brazil	Case–control	PCM: 4	752 miRNAs	Several miRNAs overexpressed (e.g., miR1323p, miR604, miR29b3p); one miRNA underexpressed (miR4233p)	-
Attia 2020 [31]	Egypt	Case–control	Sepsis: 50, Control: 20	miR-146a and miR-150	Significant correlation between miR-146a and miR-150 expression (r = 0.489, *p* < 0.001)	-
Wei 2020 [32]	China	Case–control	Sepsis: 121, Control: 60	miR-545	miR-545 significantly higher in sepsis vs. controls	AUC 0.942 for sepsis diagnosis; AUC 0.740 for 28-day mortality
Esawy 2021 [33]	Egypt	Case–control	Asthma: 30, SAFS: 30, ABPA: 30, Control: 30	miR-21 and miR-132	miR-21 elevated in all patient groups vs. controls; miR-132 highest in ABPA	ABPA vs. control: Sens 93.3%, Spec 100%; ABPA vs. asthma: Sens 90%, Spec 100%; SAFS: Sens 86.7%, Spec 80%
Fidler 2022 [34]	Hungary	Retrospective cohort	IA: 26, Control: 24	Multiple (incl. hsa-miR-191-5p, hsa-miR-106b-5p, and hsa-miR-15a-5p)	8 miRNAs downregulated in confirmed IA; 5 miRNAs had perfect discrimination (AUC 1.0)	5 miRNAs: AUC 1.0; 3 miRNAs: AUC > 0.98

### 3.3. miRNA Analyses

A total of six studies [29,30,31,32,33,34] investigating miRNAs in the context of fungal infections were identified; however, due to heterogeneity in the specific miRNAs assessed and the diversity of study designs, a meta-analysis could not be performed. Instead, a descriptive synthesis was conducted (Table 1). The included studies encompassed case–control and cohort designs, with sample sizes ranging from 8 to 181 participants, and a broad spectrum of miRNAs investigated, including miR-146a, miR-223, miR-126, miR-15b, miR-132, miR-150, miR-545, miR-21, and miR-132, as well as larger miRNA panels. Several studies demonstrated promising diagnostic performance; for example, in sepsis patients, miR-223 achieved an AUC of 0.858 (80% sensitivity, 100% specificity) and miR-146a achieved an AUC of 0.804 (63.3% sensitivity, 100% specificity), which differentiates this from systemic inflammatory response syndrome (SIRS). miR-545 showed high diagnostic accuracy for sepsis (AUC 0.942) and moderate prognostic value for 28-day mortality (AUC 0.740). In allergic bronchopulmonary aspergillosis (ABPA), miR-132 distinguished ABPA from healthy controls with 93.3% sensitivity and 100% specificity, and distinguished ABPA from allergic asthma with 90% sensitivity and 100% specificity. For invasive aspergillosis (IA), eight downregulated miRNAs, including hsa-miR-191-5p, hsa-miR-106b-5p, and hsa-miR-15a-5p, exhibited excellent discriminatory power (AUC = 1.0), with three other miRNAs exceeding an AUC of 0.98 for differentiating between proven/probable IA and non-infected controls. Collectively, these findings highlight the potential of certain miRNAs to act as highly specific and sensitive biomarkers for fungal infections. However, standardization across studies is needed before pooled quantitative estimates can be generated.

Collective evidence from these six studies highlights the growing recognition of circulating miRNAs as promising biomarkers for fungal infections and sepsis-related conditions. Although heterogeneous in design, sample size, and patient populations, these investigations converge on the utility of specific miRNAs as diagnostic and prognostic tools. Early studies focused on sepsis, where dysregulation of miR-146a, miR-150, miR-223, and miR-545 was shown to differentiate septic patients from healthy controls and cases of systemic inflammatory response syndrome (SIRS) [29,31,32]. Attia et al. [31] reported significant overexpression of miR-146a and miR-150 in septic ICU patients, correlating with infecting pathogens. By contrast, Wang et al. [29] demonstrated reduced serum levels of miR-146a and miR-223 with strong diagnostic accuracy, surpassing conventional markers such as IL-6. More recently, Wei and colleagues extended these observations by implicating miR-545 and its regulatory axis with circ-PRKCI, linking expression profiles not only to the risk of sepsis but also to severity indices and 28-day mortality [32]. Collectively, these findings underscore the fact that circulating miRNAs provide both pathogen-specific and host-response information, offering prognostic insights beyond classical biomarkers.

In fungal diseases, distinct miRNA signatures have also emerged. Esawy et al. highlighted miR-132 as a sensitive and specific marker for allergic bronchopulmonary aspergillosis (ABPA), capable of distinguishing it from severe asthma with fungal sensitization and indicating a correlation with IL-5, which is a key Th2 cytokine [33]. Similarly, Fidler et al. used high-throughput sequencing in hematology/oncology patients and identified unique miRNA signatures assoc iated with invasive aspergillosis (IA), proposing panels of up- and downregulated miRNAs as potential classifiers [34]. These studies suggest that miRNAs not only capture the immunologic milieu of fungal allergy but also detect invasive disease in immunocompromised hosts, with implications for early antifungal intervention. Beyond *Aspergillus*, Da Lacorta Singulani et al. provided preliminary evidence that patients with paracoccidioidomycosis exhibit distinctive circulating miRNA profiles, involving regulators of apoptosis and immune pathways [30]. Although based on a small cohort, these results extend the concept of fungal-associated miRNA dysregulation to endemic mycoses, supporting the broader applicability of miRNA-based diagnostics in medical mycology.

Taken together, these six studies suggest that circulating miRNAs represent a versatile biomarker platform across the spectrum of sepsis and fungal infections. Certain miRNAs, such as miR-146a, appear consistently dysregulated across different inflammatory settings, while others, like miR-132 in ABPA or specific miRNA panels in IA, display disease-specific patterns. The heterogeneity of results also reflects methodological variability, including differences in qRT-PCR versus sequencing approaches, small sample sizes, and a lack of standardized reference genes, all of which limit direct comparability. Importantly, none of the studies have yet provided prospective validation in large multicenter cohorts, nor do they fully address the challenges of integrating miRNA assays into clinical workflows.

## 4. Discussion

In this review, evidence from the analyzed literature was synthesized to derive key insights into the role of molecular biomarkers in fungal infections. The discussion is structured around four main themes:

(1) The role of miRNA and cfDNA in fungal pathogenesis;
(2) Diagnostic options;
(3) Advances in therapeutic strategies;
(4) Future directions and recommendations.

### 4.1. Role of miRNA and cfDNA in Fungal Pathogenesis

Our synthesis highlights that both miRNAs and cfDNA are closely associated with host–pathogen interactions in fungal diseases. cfDNA, originating from fungal cell death or active secretion, reflects the presence and burden of the pathogen in the bloodstream or other sterile sites. The pooled prevalence from our meta-analysis indicated that fungal cfDNA was detectable in 12% of all tested human samples, 15% of cfDNA-positive samples, and 79% of patients with confirmed fungal infections (Figure 1). These findings suggest that cfDNA levels correlate strongly with disease status and may reflect fungal load and dissemination.

Similarly, the reviewed literature demonstrates that miRNAs play a regulatory role in immune responses during fungal infections, influencing inflammation, apoptosis, and pathogen clearance. Specific miRNAs, such as miR-132, miR-223, and miR-146a, were found to be dysregulated in invasive and allergic fungal diseases. By contrast, certain miRNA panels were found to have excellent discriminatory power in differentiating confirmed invasive aspergillosis from controls (AUC values ≥ 0.98). This molecular interplay underscores the potential of miRNAs and cfDNA as complementary biomarkers in understanding fungal pathogenesis.

In host–pathogen interactions, miRNAs play a dual role, modulating immune responses, reducing inflammation, and preventing fungal survival mechanisms [35]. In fungal infections, miRNAs, such as miR-146a and miR-155, are upregulated, influencing Toll-like receptor (TLR) pathways to enhance pathogen recognition and immune activation. Conversely, miR-21 has been linked to immune evasion by suppressing pro-inflammatory cytokines such as IL-6 and TNF-α during *Candida* albicans infection, aiding in fungal persistence [21]. Other miRNAs such as miR-132-5p and miR-212-5p are involved in dendritic cell regulation during *Candida albicans* and *Aspergillus fumigatus* infections, fine-tuning immune responses through pathways like TLR and cytokine production [24]. miRNAs such as miR-145-5p, miR-424-5p, miR-99b-5p, and miR-4488 have been found upregulated in invasive aspergillosis, while others like miR-4454 and miR-7975 were downregulated in our previous study [36]. miRNA-associated pathways influence dendritic cell maturation in cryptococcosis through mechanisms such as the snhg1-miR-145a-3p-Bcl2 axis [37]. miR-132 and miR-155 have been identified as key players in enhancing immune responses to *A. fumigatus*. In *C. albicans*, miR-155 expression is specifically induced by the hyphal form, highlighting its role in distinguishing between commensal and pathogenic forms. miR-155 regulates macrophage polarization, boosting fungal clearance, while miR-132 is associated with modulating inflammation during infection [38,39]. Similarly, studies on *Aspergillus* spp. show that miRNAs like miR-223 are pivotal in immune modulation, influencing cytokine and chemokine production as a key regulator of neutrophil activation, and contributing to the clearance of *A. fumigatus* [40,41,42,43,44]. miR-146a and miR-21 have been shown to modulate the Dectin-1 signaling pathway, which is essential for recognizing fungal cell wall components such as β-glucans, indicating that miRNA-related Dectin-1 polymorphisms may influence susceptibility to fungal diseases [45,46,47,48].

Recurrently identified miRNAs across different fungal infections appear to play pivotal roles in host–pathogen interactions. Among them, miR-146a and miR-223 have been associated with immune regulation in sepsis and invasive aspergillosis, particularly through modulation of toll-like receptor signaling, neutrophil activity, and cytokine release. miR-132 has been reported in both allergic bronchopulmonary aspergillosis and invasive aspergillosis, suggesting its utility as a diagnostic biomarker in respiratory fungal diseases. Likewise, miR-155 has been linked to *Candida albicans* and *Aspergillus* infections, where it influences macrophage polarization and antifungal immunity. Importantly, our previous study [36] published in the Journal of Applied Genetics (MicroRNA expression profile of alveolar epithelial cells infected with *Aspergillus fumigatus*, 2024, 65: 627–634) demonstrated significant upregulation of miR-21, miR-186-5p, miR-490-5p, miR-26a-5p, and miR-424-5p, and downregulation of miR-145-5p, miR-583, miR-3978, and miR-4454 in *A. fumigatus*–infected epithelial cells. These findings reinforce the notion that certain miRNAs are consistently dysregulated across diverse fungal infections and may therefore serve as broad-spectrum biomarkers for diagnosis and prognosis, as well as potential targets for novel host-directed antifungal therapies. These findings, summarized in Table 2, reinforce the notion that certain miRNAs are consistently dysregulated across diverse fungal infections and may therefore serve as broad-spectrum biomarkers for diagnosis and prognosis, as well as potential targets for novel host-directed antifungal therapies.

On the other hand, cfDNA, released into the circulation through fungal cell lysis or active secretion, reflects the presence and burden of the pathogen in the host. In our meta-analysis, cfDNA was detectable in 79% of patients with proven fungal infections, indicating its strong correlation with invasive disease [49]. This biomarker not only mirrors fungal load and dissemination but can also enable species-level identification when pathogen-specific sequences are targeted. Importantly, cfDNA may offer diagnostic value in cases where conventional culture methods fail to detect the pathogen [19,24,50]. This issue will be discussed in greater detail in the following section. The relatively small sample size in this subgroup analysis (34 patients across four studies) limits statistical power and may have contributed to the wider confidence intervals observed. Therefore, the pooled estimate of 79% cfDNA positivity in proven fungal infections should be interpreted with caution.

High heterogeneity was observed in several pooled analyses (I^2^ values ranging from 80.4% to 97.3%). This heterogeneity is likely attributable to differences in study design, patient populations (e.g., immunocompromised versus immunocompetent, age related variations), assay methodologies (PCR-based versus sequencing-based), sample sizes, and geographic settings. These methodological and clinical variations should be considered when interpreting the pooled prevalence estimates (Table 3).

### 4.2. Diagnostic Options

The integration of cfDNA and miRNA analysis into diagnostic workflows offers a promising approach to improve early detection and diagnostic accuracy for fungal infections. cfDNA testing has the advantage of being culture-independent, allowing for earlier detection compared to conventional mycological methods. High cfDNA detection rates have been identified in patients with proven fungal disease, suggesting its value in diagnosing high-risk populations such as transplant recipients, ICU patients, and those with hematological malignancies.

miRNA profiling, while more heterogeneous in application, revealed high diagnostic accuracy in specific contexts. For example, miR-132 distinguished allergic bronchopulmonary aspergillosis from controls with 93.3% sensitivity and 100% specificity, while certain miRNA panels accurately discriminated invasive aspergillosis cases from non-infected controls [51]. However, diagnostic implementation is currently limited by the lack of standardized assays, their variability in miRNA targets, and differences in analytical platforms [27]. The integration of miRNAs and EVs into clinical practice offers a promising avenue for improving the diagnosis of fungal infections.

Diagnostic platforms combined with miRNA profiling demonstrated superior sensitivity and specificity compared to traditional fungal diagnosis [52]. Recent advancements in omics technologies have enabled detailed characterization of exosomal cargo, including their miRNAs, proteins, and lipids. The significant diagnostic potential of miRNAs has been explored in several studies. High-throughput sequencing identified distinct miRNA profiles in patients with invasive aspergillosis, distinguishing them from non-infected individuals [18]. miRNA biomarkers such as miR-132 and miR-125 have shown promise in diagnosing fungal infections and monitoring disease progression. Circulating miRNAs, including miR-191-5p and miR-106b-5p, have demonstrated high discriminatory power in detecting invasive aspergillosis in immunocompromised patients [18,51,52].

cfDNA-based assays are culture-independent, minimally invasive, and capable of detecting invasive fungal infections earlier than conventional mycological methods. In high-risk populations, such as patients with hematologic malignancies, solid organ transplants, or intensive care settings, cfDNA testing has demonstrated superior sensitivity and specificity compared to conventional biomarkers, including galactomannan. Its highly negative predictive value also offers the potential to reduce unnecessary antifungal exposure, thereby optimizing antifungal stewardship [53]. Importantly, this prevalence suggests that nearly one in six patients with a cfDNA-positive result harbors evidence of fungal involvement, which is a clinically meaningful rate given the challenges of diagnosing invasive mycoses. Compared with culture- or biomarker-based assays, which are limited by sensitivity, specificity, or pathogen coverage, cfDNA sequencing offers a more comprehensive approach that can simultaneously identify fungi alongside bacterial and viral pathogens. These results underscore the potential for cfDNA to be integrated into multimodal diagnostic algorithms, improving early recognition of fungal infections and guiding the timelier initiation of antifungal therapy.

Taken together, the three pooled analyses provide complementary insights into the diagnostic role of cfDNA in fungal infections. First, across more than 20,000 samples, the overall prevalence of fungal positivity in cfDNA was 12% (95% CI: 0.06–0.22), underscoring the ability of cfDNA sequencing to capture fungal DNA in a substantial subset of patients. Second, when restricted to cfDNA-positive samples, 15% of cases (95% CI: 0.10–0.22) were attributable to fungal pathogens, highlighting the incremental contribution of cfDNA to the detection of invasive mycoses within the broader landscape of infectious diseases. Finally, among patients with confirmed fungal infections, cfDNA positivity reached 79% (95% CI: 0.62–0.90), supporting the sensitivity of cfDNA sequencing in this setting, despite being based on very small cohorts. Collectively, these findings suggest that while fungi represent a minority of cfDNA-detected pathogens overall, cfDNA testing provides a clinically meaningful yield and high sensitivity in proven cases, emphasizing its potential value as a complementary tool for early and accurate diagnosis of invasive fungal infections.

### 4.3. Advances in Therapeutic Strategies

Beyond diagnosis, both cfDNA and miRNAs have potential roles in guiding therapy. cfDNA quantification could serve as a dynamic biomarker to monitor treatment response, allowing for early detection of therapeutic failure or disease recurrence. Similarly, miRNA expression profiles could provide prognostic information, such as predicting patient outcomes or identifying those at risk of progression to invasive disease [28].

Emerging therapeutic strategies should also consider targeting miRNA pathways to modulate host immune responses against fungi. Although such approaches remain in preclinical stages, studies suggest that restoring or inhibiting specific miRNAs could enhance antifungal immunity. Furthermore, integrating cfDNA and miRNA data with other biomarkers (e.g., galactomannan and β-D-glucan) could form multi-marker algorithms to optimize antifungal stewardship and minimize unnecessary drug exposure [54].

Therapeutically, miRNAs can be targeted using mimics or inhibitors to modulate host–pathogen interactions. In addition to diagnostics, transcriptomics and in silico analyses have identified miRNAs as potential therapeutic targets in infections like sporotrichosis and those caused by *Paracoccidioides* species [55]. For instance, the inhibition of miR-155 has been shown to enhance antifungal immune responses in preclinical models [39]. However, challenges such as delivery methods and off-target effects need to be addressed in a detailed manner before clinical application. Another promising approach is the use of synthetic miRNAs designed to target fungal-specific genes, which has demonstrated efficacy in laboratory settings [56].

Extracellular vesicles containing miRNAs facilitate crosstalk between immune and epithelial cells, orchestrating coordinated responses to bacterial challenges [57]. This intercellular communication underscores the complexity of miRNA-mediated immune regulation and highlights the potential of EVs as targets for therapeutic intervention. These findings suggest that targeting miRNA networks could provide a comprehensive strategy for enhancing antifungal immunity while minimizing host tissue damage. Furthermore, the ability of exosomes to selectively deliver biomolecules has increased interest in their use as therapeutic vehicles. Engineered EVs loaded with antifungal drugs have shown enhanced efficacy and reduced toxicity in preclinical models [58,59]. Engineered exosomes loaded with amphotericin B have demonstrated enhanced efficacy and reduced toxicity in preclinical models. Furthermore, mesenchymal stem cell-derived EVs exhibit immunomodulatory properties, which can be harnessed to treat fungal infections [54]. Overall, EVs modified to overexpress specific miRNAs have shown promising results in modulating immune responses against fungal pathogens. The concept of “synthetic biology” is also gaining traction, where engineered miRNA circuits are being developed to precisely regulate gene expression in host cells during fungal infections. These synthetic constructs can be programmed to sense fungal biomarkers and initiate therapeutic responses, representing a paradigm shift in antifungal therapy [50]. Immunomodulation through miRNAs and EVs holds significant potential. Enriched EVs with miRNA mimics have shown efficacy in preclinical models of fungal infections by enhancing host immune responses and reducing inflammation [60]. Another area of interest is the use of EVs as vaccine platforms, enhancing their ability to present fungal antigens to the host immune system and elicit protective responses. Their potential in vaccine development, particularly against fungal and viral infections, is gaining recognition. By exploiting their natural role in communication and cargo delivery, researchers aim to harness exosomes for targeted immunotherapies and innovative treatments. For example, dendritic cell-based vaccines utilizing EVs have emerged as innovative strategies to boost antifungal immunity [60,61].

Serial quantification of cfDNA provides an objective tool for monitoring responses to antifungal treatment. A rapid decline in cfDNA levels early in therapy is often indicative of treatment success, whereas persistent or rising levels may signal therapeutic failure or disease recurrence. This approach has the potential to personalize treatment duration, minimizing toxicity and avoiding premature discontinuation of therapy [62].

However, several challenges remain. Specifically, standardization of miRNA and cfDNA isolation techniques, understanding their mechanisms of action, and addressing safety concerns are critical for translating these findings into clinical applications. Moreover, the cost-effectiveness and scalability of miRNA- and EV-based diagnostics and therapeutics need to be evaluated.

Despite encouraging results, significant heterogeneity exists among studies. Sample sizes were generally small, ranging from exploratory cohorts of fewer than 10 patients in PCM to moderate case–control designs in sepsis and ABPA. Methodological variability was also evident: while some studies employed targeted qRT-PCR for candidate miRNAs, others used high-throughput sequencing. Differences in normalization strategies, reference gene selection, and assay sensitivity limited the comparability of findings. Furthermore, external validation in multicenter cohorts was lacking, and none of the studies addressed how miRNA testing could be integrated into existing diagnostic algorithms.

### 4.4. Future Directions and Recommendations

To translate the promising potential of cfDNA and miRNAs into clinical practice, several key steps are required as follows:

Standardization of Methodology: Harmonizing pre-analytical and analytical protocols for cfDNA extraction, quantification, and miRNA profiling is essential to ensure reproducibility across laboratories.

Large-Scale Validation Studies: Multicenter prospective studies are needed to validate diagnostic and prognostic performance in diverse patient populations, including pediatric, immunocompromised, and ICU cohorts.

Integration with Existing Diagnostic Algorithms: Combining molecular biomarkers with established diagnostic tools could improve sensitivity and specificity, particularly in early or subclinical infections.

Cost-Effectiveness and Feasibility Assessments: Economic analyses and operational evaluations will determine the practicality of routine implementation in different healthcare settings.

Exploration of Therapeutic Modulation: Preclinical and translational studies should investigate whether modulating specific miRNAs or monitoring cfDNA kinetics can directly impact patient outcomes.

## 5. Conclusions

In conclusion, cfDNA and miRNAs represent promising molecular biomarkers with significant potential to advance the understanding, diagnosis, and management of fungal infections. Current evidence underscores their diagnostic and therapeutic promise; however, routine clinical implementation will require rigorous standardization of isolation methods, validation across diverse patient populations, and integration into established clinical pathways. Advances in nanotechnology and synthetic biology may further enhance the therapeutic applications of exosomes and miRNAs, offering innovative strategies to combat fungal diseases. At the same time, cfDNA sequencing provides a sensitive and comprehensive platform for early detection, complementing conventional diagnostics. Future research should prioritize large-scale clinical trials to establish efficacy, safety, and reproducibility, while also ensuring adherence to ethical standards. Collaborative efforts among researchers, clinicians, and industry stakeholders will be pivotal in unlocking the full potential of cfDNA- and miRNA-based approaches, ultimately bridging the gap between basic science and clinical practice in managing fungal infections.

## Figures and Tables

**Table 2:** Summary of reported microRNAs (miRNAs) in fungal infections, including the associated disease context, regulation status (upregulated or downregulated), and their reported or predicted biological functions. The table integrates evidence from clinical and experimental studies, highlighting miRNAs repeatedly identified across multiple fungal infections (e.g., miR-146a, miR-223, miR-132, miR-155). These shared miRNAs may represent broad-spectrum biomarkers and potential targets for modulating host immune responses in fungal diseases.

miRNA	Disease/Condition	Regulation	Reported/Predicted Function
miR-146a	Sepsis, Invasive aspergillosis	Downregulated	Regulates TLR signaling, controls inflammation and pathogen recognition
miR-223	Sepsis, Aspergillosis	Downregulated	Neutrophil activation, cytokine regulation, pathogen clearance
miR-132	ABPA, Invasive aspergillosis	Upregulated	Th2 immune response, inflammation modulation
miR-155	*Candida albicans*, *Aspergillus* spp.	Upregulated	Macrophage polarization, enhances fungal clearance
miR-21	ABPA, Asthma, Aspergillosis (in vitro)	Upregulated	Suppresses IL-6/TNF-α, promotes immune evasion
miR-545	Sepsis	Upregulated	Associated with severity and mortality prediction
miR-191-5p, miR-106b-5p, miR-15a-5p	Invasive aspergillosis (hematology/oncology patients)	Downregulated	High discriminatory power for diagnosis in immunocompromised patients
miR-21-5p, miR-145-5p, miR-583, miR-3978, miR-4488, miR-4454	*Aspergillus fumigatus* infection (A549 in vitro)	Downregulated	Differential expression associated with host response in epithelial cells
miR-186-5p, miR-490-5p, miR-26a-5p, miR-26b-5p, miR-424-5p, miR-548d-3p, miR-196a-5p, miR-150-5p, miR-17-5p, miR-99b-5p	*Aspergillus fumigatus* infection (A549 in vitro)	Upregulated	Differential expression associated with host response in epithelial cells

**Table 3:** Risk of bias and study quality assessment of the studies included in the systematic review. Diagnostic accuracy studies were evaluated using the QUADAS-2 tool, while observational and cohort studies were assessed with the Newcastle–Ottawa Scale (NOS) and Joanna Briggs Institute (JBI) Critical Appraisal Checklist for Case Series. The majority of studies demonstrated low to moderate risk of bias, with limitations mainly related to small sample sizes, retrospective design, and lack of standardized assays. Overall, study quality was sufficient to support synthesis, but methodological variability should be considered when interpreting the pooled estimates. IFD: Invasive fungal disease, ID: Infectious Disease.

Study (Author, Year)	Design	Tool Used	Risk of Bias/Quality Score	Comments
Armstrong, 2019 [28]	Prospective cohort (IFD)	QUADAS-2	Moderate risk	Small sample size, Standardized test protocol
Balks, 2025 [23]	Prospective cohort (Sepsis)	QUADAS-2	Low risk	Clear patient selection; appropriate index test
Chen, 2024 [21]	Prospective cohort (Suspected infectious diseases)	QUADAS-2	Moderate risk	Sample location differences, temporal variations in treatment protocols
Gracia, 2024 [17]	Case series (Mucormycosis)	QUADAS-2	Moderate risk	Small sample size, Clear patient selection
Hogan, 2021 [24]	Retrospective cohort (ID)	QUADAS-2	Low risk	Standardized test protocol, Multicenter
Hong, 2018 [27]	Retrospective (IFD)	QUADAS-2	Moderate risk	Small sample size, Standardized test protocol
Kushner, 2024 [16]	Retrospective (IFD)	QUADAS-2	Low risk	Clear patient selection; appropriate index test
Pang, 2024 [26]	Retrospective cohort (ID)	QUADAS-2	Low risk	Clear patient selection; Multicenter
Park, 2023 [20]	Descriptive (ID)	STROBE	Moderate/High Reporting Quality	Standardized test, Multicenter, Big sample size
Shah, 2024 [25]	Retrospective (ID)	NOS	6/9 stars	Clear patient selection, Standardized test
Tang, 2025 [22]	Cross-sectional (ID)	QUADAS-2	Low risk	Standardized test, Clear sample selection
Vissichelli, 2023 [15]	Retrospective observational study (ID)	NOS	6/9 stars	Limited sample size, Sample location differences
Wang, 2021 [14]	Prospective cohort (Sepsis)	QUADAS-2	Low risk	Strong design, Clear sample selection
Weiss, 2023 [19]	Retrospective cohort (ID)	JBI	Moderate risk	Small sample size, appropriate index test
Xu, 2023 [18]	Retrospective cohort (ID)	QUADAS-2	Low risk	Clear patient selection; appropriate index test
Zhang, 2024 [12]	Retrospective cohort (ID)	QUADAS-2	Moderate risk	
Wang, 2010 [29]	Case–control (Sepsis)	QUADAS-2	Low risk	Clear patient selection; appropriate index test
Attia, 2020 [31]	Case–control (Sepsis)	QUADAS-2	Moderate risk	Small sample size; unclear blinding
Wei, 2020 [32]	Case–control (Sepsis)	NOS	7/9 stars	Good selection and comparability; limited outcome follow-up
Esawy, 2021 [33]	Case–control (ABPA)	NOS	6/9 stars	Adequate selection; limited sample size
Fidler, 2022 [34]	Retrospective cohort (IA)	NOS	8/9 stars	Strong design; potential retrospective bias
Da Lacorta Singulani, 2017 [30]	Case–control (PCM)	NOS	5/9 stars	Very small sample; exploratory findings

## Data Availability

No new data were created or analyzed in this study. Data sharing is not applicable to this article.

## Abbreviations

The following abbreviations are used in this manuscript:

miRNA	Micro RNA
cfDNA	Cell free DNA
MVBs	Multivesicular bodies
HSP	Heat shock protein
PRISMA	Preferred Reporting Items for Systematic Reviews and Meta-Analyses
PROSPERO	Prospective Register of Systematic Reviews
CD	Cluster of differentiation
IFD	Invasive fungal disease
ID	Infectious Disease
NOS	Newcastle–Ottawa Scale
JBI	Joanna Briggs Institute (JBI) Critical Appraisal Checklist
